# How to choose and interpret similarity indices to quantify the variability in gait joint kinematics

**DOI:** 10.1080/23335432.2018.1426496

**Published:** 2018-02-01

**Authors:** Roberto Di Marco, Emilia Scalona, Alessandra Pacilli, Paolo Cappa, Claudia Mazzà, Stefano Rossi

**Affiliations:** a Department of Mechanical Engineering and INSIGNEO Institute for in silico Medicine, The University of Sheffield, Sheffield, UK; b Department of Mechanical and Aerospace Engineering, ‘Sapienza’ University of Rome, Rome, Italy; c Department of Economics, Engineering, Society and Business Organization (DEIM), University of Tuscia, Viterbo, Italy

**Keywords:** Variability indices, repeatability, reproducibility, joint kinematics simulations, gait analysis

## Abstract

Repeatability and reproducibility indices are often used in gait analysis to validate models and assess patients in their follow-up. When comparing joint kinematics, their interpretation can be ambiguous due to a lack of understanding of the exact sources of their variations. This paper studied four indices (Root Mean Square Deviation, Mean Absolute Variability, Coefficient of Multiple Correlation, and Linear Fit Method) in relation to five confusing-factors: joints’ range of motion, sample-by-sample amplitude variability, offset, time shift and curve shape. A first simulation was conducted to test the mathematics behind each index. A second simulation tested the influence of the curve shape on the indices using a Fourier’s decomposition. The Coefficient of Multiple Correlation and the Linear Fit method Coefficients were independent from the range of motion. Different Coefficients of Multiple Correlation were found among different joints, leading to misinterpretation of the results. The Linear Fit Method coefficients should not be adopted when time shift increases. Root Mean Square Deviation and Mean Absolute Variability were sensitive to all the confusing-factors. The Linear Fit Method coefficients seemed to be the most suitable to assess gait data variability, complemented with Root Mean Square Deviation or Mean Absolute Variability as measurements of data dispersion.

## Introduction

Human joint kinematics and dynamics assessed with 3D gait analysis have been proven to be suitable for clinical decision-making, thanks also to repeatability and reproducibility studies that validate relevant measurements and modelling techniques (Carson et al. 2001; Arnold et al. 2013; Benedetti et al. 2013; Leigh et al. 2014). According to metrological standards (JCGM 2012), the repeatability is the measurement precision associated with the same operator performing the same procedure on the same group of subjects that in gait analysis quantifies the within- and between-subject variability. The reproducibility, instead, is the measurement precision associated with different operators performing the same procedure on the same group of subjects that quantifies the between-operator variability of the data. Several indices have been proposed and used as summarised in (Chau 2001a, 2001b) with some of these, including standard deviation (SD), coefficient of variation (CV), Intraclass Correlation Coefficient (ICC) (Shrout and Fleiss 1979), Standard Error of Measurement (SEM) (Stratford and Goldsmith 1997), Technical Error of Measurement (TEM) (Curtis et al. 2009), and Minimum Detectable Changes (MDC) (Klejman et al. 2010), have been used to quantify the data dispersion around the reference value at specific instants of the gait cycle. Other indices, instead, including the Root Mean Square Deviation (RMSD) (Picerno et al. 2008), Mean Absolute Variability (MAV) (Ferrari et al. 2010), Coefficient of Multiple Correlation (CMC) (Kadaba et al. 1989), and the Linear Fit Method (LFM) coefficients (Iosa et al. 2014), have been used to describe the whole within-stride variability, needed to quantify the similarity of the curve patterns along the whole gait cycle.

Repeatability and reproducibility indices (RI) are influenced by various factors, which lead to limited interpretation of the relevant results. These factors, here indicated as confusing-factors, are: (*a*) the range of motion of the considered joint (Steinwender et al. 2000); (*b*) the sample by sample amplitude variations from the averaged pattern, which is typical for each joint and representative for the within-subject variability (Winter 1984); (*c*) the offset among curves, mostly depending on marker repositioning (Leardini et al. 1999); (*d*) the time shift due to physiological and/or pathological gait phases variability (Mileti et al. 2016); and (*e*) the different curve shapes among joints and planes (Røislien et al. 2012). The effects of each confusing-factor on the RI have not been tested, and a comparative analysis that aims to clearly interpret the relationship between RI and the confusing-factors is still lacking in literature. Therefore, this research aims to fill this gap via simulations on both synthetic and experimental data gathered from healthy adults, providing also a guide on how to choose the most suitable repeatability and reproducibility indices, and how to interpret the results when dealing with joint kinematic curves.

## Materials and methods

To test the mathematical formulation of the indices, tests were initially conducted on generic *sine*-*curve* data, which were parametrised according to the aforementioned confusing-factors. This allows to easily impose changes to one factor at a time, while leaving the curve shape unvaried, and to observe the relevant variations in the RI values. Then, to test the effect of changing the shape of the curves, keeping the focus on gait analysis applications, sagittal hip, knee and ankle joint kinematics gathered from experimental data were decomposed with a Fourier’s analysis. The so obtained Fourier’s coefficients were then modified to simulate the confusing-factors on the joint kinematics (*Fourier-based* data).

### Experimental setup

Ten healthy adults (males, age: 27.0 ± 1.9 years, body mass: 76.7 ± 13.8 kg, leg length: 85.3 ± 4.6 cm), with no reported pathologies influencing their walking, were enrolled in this study after having signed an informed consent form (ethical approval granted by The University of Sheffield).

One operator performed the marker placement on the right lower limb of each participant (Di Marco et al. 2016). Gait data were recorded with a 10-camera stereophotogrammetric system (T-160, 100 Hz, Vicon Nexus 1.8.5, Vicon Motion System Ltd – Oxford, UK). Pre-processing was conducted within Nexus (smoothing with a Woltring routine, size 30 (Woltring 1986)).

Participants walked barefoot for two minutes on a treadmill (ADAL3D-F, TECMACHINE HEF Groupe – France) at their self-selected speed, which was chosen during the first test session (0.82 ± 0.15 m/s). Two experimental sessions were performed one month apart, and five right strides were retained for the analysis. During the second session, the treadmill was set at the same speed of the first data collection session. Sagittal lower limb kinematics (available at dx.doi.org/10.15131/shef.data.3502712 (Di Marco et al. 2016)) were extracted and post-processed within MATLAB (R2015b, The MathWorks, Inc. – Natick, MA, USA).

The ranges of variation and the magnitude of variation of: joint range of motion (ROM), joint ROM fluctuations (*α*), offset between curves (*O*), and time shift (*τ*) were calculated from the within- and the between-subject analyses performed on the experimental data. The obtained ranges of variations were then used for the *Sine*-*curve* and the *Fourier-based* data simulations.

### Repeatability and reproducibility indices

Four indices that attempt to quantify the data similarity over the whole gait cycle were considered: RMSD, MAV, CMC and LFM coefficients. RMSD represents the root square of the variance, evaluated sample by sample, between the curves and the averaged curve over the gait cycle (JCGM 2008a, 2012). Similarly, MAV measures the average of the sample by sample difference between maximum and minimum values among the compared curves (Ferrari et al. 2010; Palermo et al. 2014). CMC is the widest used index to evaluate the repeatability of waveforms and it represents the root square of the adjusted coefficient of multiple determination as reported in (Kadaba et al. 1989). CMC is expected to return values between 0 and 1, and can be stratified as: (i) ‘poor similarity’ when 0 < CMC < 0.60; (ii) ‘moderate similarity’ when 0.60 ≤ CMC < 0.75; (iii) ‘good similarity’ when 0.75 ≤ CMC < 0.85, (iv) ‘very good similarity’ when 0.85 ≤ CMC < 0.95; and (v) ‘excellent’ when 0.95 ≤ CMC ≤ 1 (Garofalo et al. 2009). LFM calculates the linear regression between a set of curves and a reference averaged curve, returning separate information about the scaling factor (*a*_1_), the weighted averaged offset (*a*_0_), and the trueness of the linear relation between them (*R*^2^). When *R*^2^ > 0.5, the assumption of linearity is considered valid, and *a*_1_ and *a*_0_ can be interpreted as meaningful (Iosa et al. 2014). The coefficients *a*_1_ and *a*_0_ tend to their ideal values (i.e. 1 and 0, respectively) when comparing *n* curves with their averaged pattern (Iosa et al. 2014). Thus, to have a measure of the variations, it is worthy to report and observe the standard deviations for both *a*_1_ and *a*_0_ (SD − *a*_1_ and SD − *a*_0_).

### Data analysis

#### Sine-curve data

Following the methodology proposed in (Røislien et al. 2012), groups of five curves ($k_{j}\left(t\right)$ with *j* is the number of the simulated strides from 1 to 5 and *t* is the number of the time samples, i.e. 100) were generated from the following mathematical model:

(1) $$k_{j}\left(t\right)=O+\left(1+\alpha \right)\frac{\mathrm{ROM}}{1.76}\left[0.5\sin\frac{2\pi \left(t-\tau \right)}{100}+0.5\sin\frac{4\pi \left(t-\tau \right)}{100}\right]$$

where *ROM*, *α*, *O* and *τ* are the previously described parameters. To obtain the desired *ROM* to be imposed, the amplitude of the sine-terms in Equation (1) was normalised by dividing term in the square brackets by its amplitude. Groups of five curves, equally spaced with respect to the single confusing-factor, were obtained by modifying each parameter once at time, generating four data-sets. *ROM* was set equal to 5° when varying *α*, *O* and *τ*. The imposed values of the confusing-factors, as well as the magnitude of their variations, were calculated as described in the Experimental setup section and are shown in Table 1. Examples of the generated curves are available in the Supplementary materials.

**Table 1.** Variations imposed in the simulations based on the *sine*-*curve* for: (1) amplitude (*ROM*); (2) amplitude variability (*α*); (3) offset (*O*); and (4) time shift (*τ*). Each case (i.e. each row) represents single set of 5 curves, equally spaced with respect to the single confusing-factor

		*ROM* (°)	*α* (%_ROM_)	*O* (%_ROM_)	*τ* (%_GaitCycle_)
Case 1	*I*	5	±2.5	0	0
	*II*	15	±2.5	0	0
	*III*	30	±2.5	0	0
	*IV*	40	±2.5	0	0
	*V*	50	±2.5	0	0
	*VI*	60	±2.5	0	0
Case 2	*I*	5	±2.5	0	0
	*II*	5	±5.0	0	0
	*III*	5	±7.5	0	0
	*IV*	5	±10.0	0	0
	*V*	5	±12.5	0	0
	*VI*	5	±15.0	0	0
Case 3	*I*	5	0	±5	0
	*II*	5	0	±20	0
	*III*	5	0	±40	0
	*IV*	5	0	±60	0
	*V*	5	0	±80	0
	*VI*	5	0	±100	0
Case 4	*I*	5	0	0	0–5
	*II*	5	0	0	0–10
	*III*	5	0	0	0–15
	*IV*	5	0	0	0–20
	*V*	5	0	0	0–25
	*VI*	5	0	0	0–30

The four selected RI were then calculated for each group of the generated curves. For RMSD and LFM, each *j*th curve was compared to the mean of the five curves from the same group, taken as a reference value.

#### Fourier-based data

Fourier decomposition (Equation (2)) was performed analysing the averaged sagittal hip, knee and ankle kinematics obtained from all the participants. The Fourier decomposition of each mean curve is:

(2) $$y\left(t\right)=\frac{A_{0}}{2}+\sum_{k=1}^{n}\left[A_{k}\cos\left(\mathrm{kt}\right)+B_{k}\sin\left(\mathrm{kt}\right)\right],\;t\;\text{from}\;0\;\text{to}100$$

**Figure 1.**
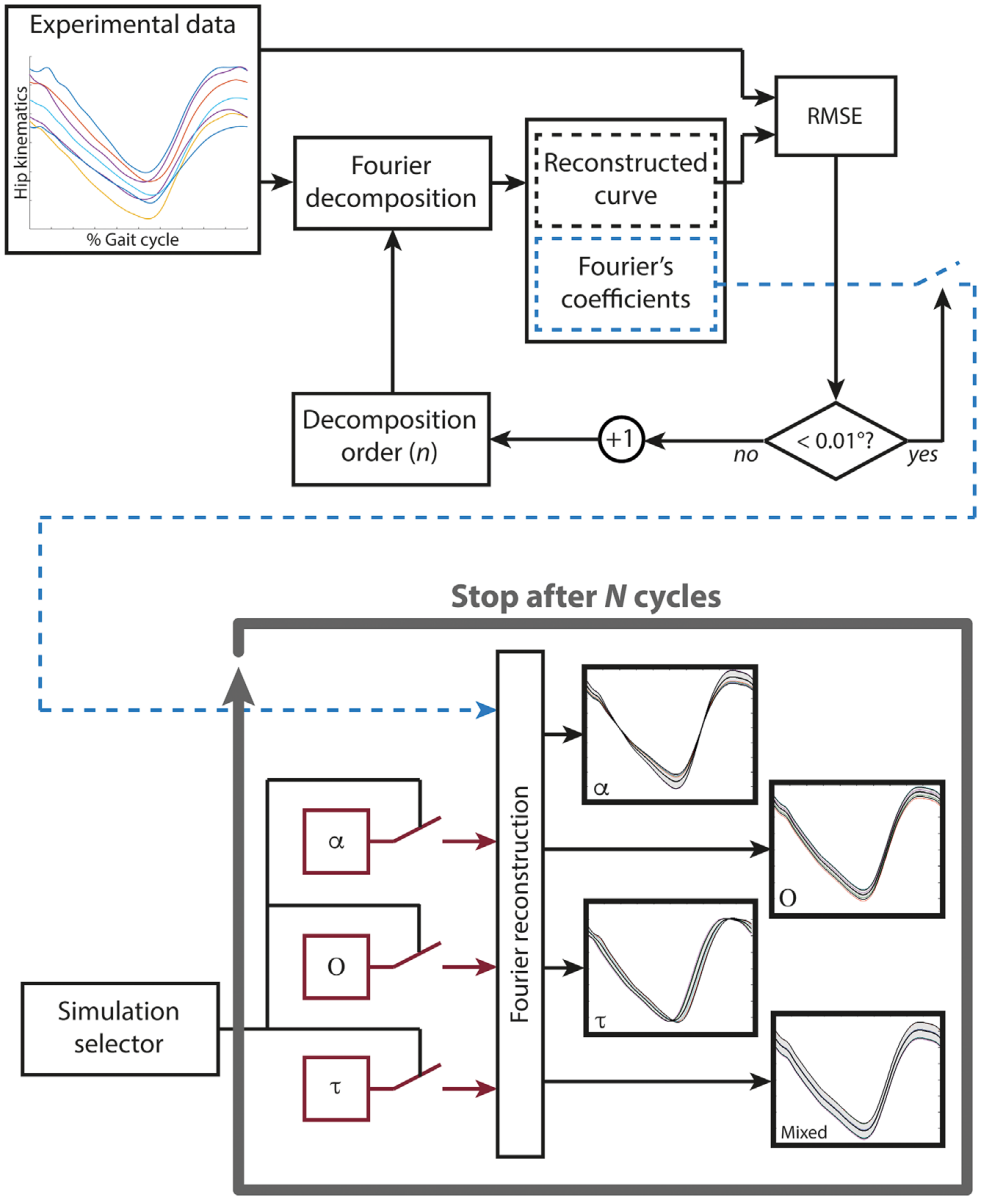
Simulation procedure based on a Fourier’s decomposition of the lower limb joint kinematics gathered from a sample of healthy adults (*Fourier*-*based data*). In the present study the number of simulations (*N*) was set equal to 1000

The decomposition order (*n*) was stopped when the RMSE between the averaged pattern and the curve reconstructed with the Fourier series was lower than 1/100 of the precision of the technical measurement procedure (1° (Della Croce et al. 1999)). Three simple simulations were obtained changing the Fourier coefficients (*A*_0_, *A*_*k*_ and *B*_*k*_) in order to simulate the variation of *α*, *O*, and *τ* on the curves. A mixed simulation (MS), accounting for all the previous parameters, was then performed to verify whether it is possible to resolve the different confusing-factors among curves. Specifically, a Monte Carlo procedure was used to perform 1000 simulations, generating groups of five curves for each simulation (JCGM 2008b). A uniform probability density function was considered for *α*, *O*, and *τ*, whose ranges of variations were chosen based on the experimental data, accounting for both within- (*WS*) and between-subjects (*BS*) variability (Table 2). Further details are available in the Supplementary materials. Finally, averaged values and standard deviations for CMC and MAV among the values obtained from the 1000 simulations were calculated for the *WS* and *BS* analyses. Whereas, the averages and the standard deviations of the LFM coefficients, and RMSD were firstly calculated among the five curves of each group. Then, the average among the 1000 simulations of the obtained averages and standard deviations were reported as results for the LFM coefficients and RMSD. The adopted procedure is summarised in Figure 1.

**Table 2.** Maximum range of variations imposed to amplitude variability (*α*), offset (*O*), and time shift (*τ*) for the simulations performed on *Fourier*-*based data*

	*Within*-*subjects* (WS)			*Between*-*subjects* (BS)		
	*α (%*_*ROM*_ *)*	*O (%*_*ROM*_ *)*	*τ (%*_*GaitCycle*_ *)*	*α (%*_*ROM*_ *)*	*O (%*_*ROM*_ *)*	*τ (%*_*GaitCycle*_ *)*
Hip	5	5	5	10	30	10
Knee	5	5	5	5	15	10
Ankle	5	5	5	10	20	10

## Results

### Sine-curve data

Table 3 shows the results obtained for the simulations on the *sine*-*curve* data. When varying *ROM* (Case 1), as the *ROM* increased, the distances between the generated curves increased and consistently did MAV and RMSD, whereas CMC and the LFM coefficients did not detect these changes. CMC, *a*_0_, and *R*^2^ did not notably change with the variations of *α* (Case 2), whereas standard deviation of *a*_1_, MAV and RMSD increased. Increasing the offset between the curves *O* (Case 3), CMC dramatically decreased from >0.99 to a complex value, indicating a complete loss of correlation among the compared curves. The standard deviation of *a*_0_ increased with the imposed *O*, whereas *a*_1_ and *R*^2^ reached their ideal values (i.e. 1). MAV returned exactly the maximum imposed *O*, and the mean and standard deviation of RMSD values increased. The variation of the time shift *τ* (Case 4) highlighted a decrease in both CMC and *R*^2^, and consequently the coefficients *a*_1_ and *a*_0_ were not further interpreted. MAV and RMSD increased with *τ*.

**Table 3.** Values of coefficient of multiple correlation (CMC), linear fit method (LFM) coefficients, mean absolute variability (MAV) and root mean square deviation (RMSD) obtained from the simulations performed on the *sine*-*curve*, changing its amplitude (*ROM*), amplitude variability (*α*), offset (*O*) and time shift (*τ*)

			CMC	LFM coefficients			MAV (°)	RMSD (°)
				*a*_1_	*a*_0_ (°)	*R* ^2^		
Case 1	ROM (°)	*I*	>0.99	1.00 ± 0.04	0.0 ± 0.0	1.00 ± 0.00	0.1	0.0 ± 0.0
		*II*	>0.99	1.00 ± 0.04	0.0 ± 0.0	1.00 ± 0.00	0.3	0.1 ± 0.1
		*III*	>0.99	1.00 ± 0.04	0.0 ± 0.0	1.00 ± 0.00	0.7	0.3 ± 0.2
		*IV*	>0.99	1.00 ± 0.04	0.0 ± 0.0	1.00 ± 0.00	0.9	0.3 ± 0.2
		*V*	>0.99	1.00 ± 0.04	0.0 ± 0.0	1.00 ± 0.00	1.1	0.4 ± 0.3
		*VI*	>0.99	1.00 ± 0.04	0.0 ± 0.0	1.00 ± 0.00	1.3	0.5 ± 0.4
Case 2	*α* (%_ROM_)	*I*	>0.99	1.00 ± 0.04	0.0 ± 0.0	1.00 ± 0.00	0.1	0.0 ± 0.0
		*II*	>0.99	1.00 ± 0.08	0.0 ± 0.0	1.00 ± 0.00	0.2	0.1 ± 0.1
		*III*	>0.99	1.00 ± 0.12	0.0 ± 0.0	1.00 ± 0.00	0.3	0.1 ± 0.1
		*IV*	>0.99	1.00 ± 0.16	0.0 ± 0.0	1.00 ± 0.00	0.5	0.2 ± 0.1
		*V*	>0.99	1.00 ± 0.20	0.0 ± 0.0	1.00 ± 0.00	0.6	0.2 ± 0.2
		*VI*	0.99	1.00 ± 0.24	0.0 ± 0.0	1.00 ± 0.00	0.7	0.3 ± 0.2
Case 3	*O* (%_ROM_)	*I*	>0.99	1.00 ± 0.00	0.0 ± 0.2	1.00 ± 0.00	0.5	0.2 ± 0.1
		*II*	0.87	1.00 ± 0.00	0.0 ± 0.8	1.00 ± 0.00	2.0	0.6 ± 0.4
		*III*	0.61	1.00 ± 0.00	0.0 ± 1.6	1.00 ± 0.00	4.0	1.2 ± 0.8
		*IV*	0.37	1.00 ± 0.00	0.0 ± 2.4	1.00 ± 0.00	6.0	1.8 ± 1.3
		*V*	0.04	1.00 ± 0.00	0.0 ± 3.2	1.00 ± 0.00	8.0	2.4 ± 1.7
		*VI*	–	1.00 ± 0.00	0.0 ± 4.0	1.00 ± 0.00	10.0	3.0 ± 2.1
Case 4	*τ* (%_GaitCycle_)	*I*	0.98	1.00 ± 0.01	0.0 ± 0.0	0.97 ± 0.03	0.6	0.2 ± 0.1
		*II*	0.92	1.00 ± 0.06	0.0 ± 0.0	0.89 ± 0.10	1.2	0.4 ± 0.3
		*III*	0.84	1.00 ± 0.13	0.0 ± 0.0	0.77 ± 0.20	1.7	0.6 ± 0.3
		*IV*	0.73	1.00 ± 0.23	0.0 ± 0.0	0.65 ± 0.28	2.1	0.8 ± 0.4
		*V*	0.60	1.00 ± 0.35	0.0 ± 0.0	0.53 ± 0.33	2.5	1.0 ± 0.3
		*VI*	0.46	1.00 ± 0.47	0.0 ± 0.0	0.43 ± 0.33	2.8	1.1 ± 0.3

Note:

– has to be intended as the method has given complex values.

### Fourier-based data

The obtained results testing the RI on the *Fourier*-*based* data are shown in Table 4. When varying *α*, means and standard deviations of CMC displayed slightly different values among the joints. This was more evident looking at the CMC-WS for Hip (0.99 ± 0.01), Knee (0.99 ± 0.01), and Ankle (0.98 ± 0.03). The SD − *a*_1_ changed with *α* for each joint, whereas the $\overset{¯}{R^{2}}$ always reached its ideal value with null SD − *R*^2^.

**Table 4.** Values of coefficient of multiple correlation (CMC), linear fit method (LFM) coefficients, mean absolute variability (MAV) and root mean square deviation (RMSD) obtained from the simulations performed on the *Fourier*-*based data*, changing amplitude variability (*α*), offset (*O*) and time shift (*τ*) of the curves

	Joints		ROM (°)	CMC	LFM coefficients						MAV (°)	RMSD	
					${\overset{¯}{a}}_{1}$	$\text{SD -}\;a_{1}$	${\overset{¯}{a}}_{0}$ (°)	$\text{SD -}\;a_{0}$ (°)	$\overset{¯}{R^{2}}$	$\text{SD -}\;R^{2}$		$\bar{\text{RMSD}}$ (°)	SD − RMSD (°)
*α* (%_ROM_)	*Hip*	*WS*	30 ± 2	0.99 ± 0.01	1.00	0.09	0	1	1.00	0.00	2 ± 1	1	0
		*BS*	43 ± 6	0.98 ± 0.02	1.00	0.10	0	2	1.00	0.00	6 ± 4	2	1
	*Knee*	*WS*	64 ± 6	0.99 ± 0.01	1.00	0.08	0	2	1.00	0.00	6 ± 5	2	1
		*BS*	64 ± 6	0.99 ± 0.01	1.00	0.08	0	2	1.00	0.00	6 ± 5	2	1
	*Ankle*	*WS*	14 ± 1	0.98 ± 0.03	1.00	0.12	0	0	1.00	0.00	1 ± 1	0	0
		*BS*	19 ± 1	0.99 ± 0.02	1.00	0.10	0	0	1.00	0.00	1 ± 1	0	0
*O* (%_ROM_)	*Hip*	*WS*	31 ± 0	>0.99	1.00	0.00	0	1	1.00	0.00	3 ± 1	1	1
		*BS*	31 ± 0	0.90 ± 0.05	1.00	0.00	0	5	1.00	0.00	12 ± 3	4	2
	*Knee*	*WS*	51 ± 0	>0.99	1.00	0.00	0	2	1.00	0.00	4 ± 1	1	1
		*BS*	51 ± 0	0.96 ± 0.02	1.00	0.00	0	4	1.00	0.00	11 ± 3	3	2
	*Ankle*	*WS*	18 ± 0	>0.99	1.00	0.00	0	1	1.00	0.00	1 ± 0	0	0
		*BS*	18 ± 0	0.91 ± 0.04	1.00	0.00	0	2	1.00	0.00	5 ± 1	2	1
*τ* (%_GaitCycle_)	*Hip*	*WS*	31 ± 0	0.99 ± 0.01	1.00	0.01	0	0	0.99	0.01	3 ± 1	1	1
		*BS*	31 ± 0	0.97 ± 0.01	1.00	0.02	0	0	0.95	0.04	5 ± 1	2	1
	*Knee*	*WS*	51 ± 0	0.99 ± 0.01	1.00	0.01	0	0	0.98	0.02	5 ± 1	2	1
		*BS*	51 ± 0	0.95 ± 0.02	1.00	0.04	0	0	0.92	0.07	9 ± 2	4	2
	*Ankle*	*WS*	18 ± 0	0.97 ± 0.01	1.00	0.02	0	0	0.96	0.04	2 ± 1	1	1
		*BS*	18 ± 0	0.91 ± 0.03	1.00	0.07	0	0	0.87	0.11	4 ± 1	2	1
MS	*Hip*	*WS*	34 ± 1	0.99 ± 0.03	1.00	0.01	0	0	0.99	0.01	2 ± 1	1	1
		*BS*	45 ± 5	0.97 ± 0.01	1.00	0.02	0	0	0.96	0.04	7 ± 3	3	2
	*Knee*	*WS*	51 ± 1	0.98 ± 0.01	1.00	0.01	0	0	0.97	0.02	5 ± 2	3	1
		*BS*	52 ± 1	0.95 ± 0.02	1.00	0.04	0	0	0.92	0.08	10 ± 3	5	3
	*Ankle*	*WS*	17 ± 1	0.97 ± 0.01	1.00	0.02	0	0	0.96	0.04	2 ± 0	1	0
		*BS*	18 ± 1	0.91 ± 0.03	1.00	0.07	0	0	0.87	0.11	4 ± 1	2	1

Note:

*MS* stands for the simulations performed mixing the effects of *α, O,* and *τ*. *WS* and *BS* address the within- and between-subject analysis, respectively.

Comparing the within- and between-subjects, CMC decreased more explicitly when increasing the offset: e.g. for the hip, CMC-WS was higher than 0.99, whereas CMC-BS was equal to 0.90 ± 0.05. Even though less evident than in the *sine*-*curve* data, the SD − *a*_0_ varied with the imposed *O*, whereas ${\overset{¯}{a}}_{1}$ and $\overset{¯}{R^{2}}$ reached their ideal values with null standard deviations.

Coherently with the results obtained in the *sine*-*curve* data, the increment in the imposed time shift from 5% (WS) to 10% (BS) resulted in a decrease in the CMC values for all joints and both comparisons. Concerning the LFM coefficients, $\overset{¯}{R^{2}}$ decreased and SD − *R*^2^ increased with the increase of *τ*, and the even lower values were found for the BS comparison of the ankle joint ($\overset{¯}{R^{2}}$ = 0.87 and SD − *R*^2^ = 0.11).

The mixed simulation (MS) from WS to BS provided similar results of those obtained via the time shift simulation. Comparing the within- and between-subjects, MAV and RMSD increased following the increment of all the imposed variations.

## Discussion

This study presented a comparative analysis of four indices used to assess gait data repeatability and reproducibility, aiming to differentiate the effect of the defined confusing-factors (i.e. joint range of motion (*ROM*), joint ROM fluctuations (*α*), offset between curves (*O*), time shift (*τ*), curve pattern). To this purpose, the sensitivity of the RI to each of the confusing-factors has been highlighted using two simulated data-sets. The first data-set is based on simulations conducted on *sine*-*curves* aiming to test the mathematical formulation of the indices. The second one is based on gait data reconstructed via a Fourier’s decomposition of sagittal lower limb kinematics of healthy adults.

CMC was insensitive to the range of motion as it did not change when varying only the ROM of the *sine*-*curve* from 5° to 60° (Table 3). Small variations in CMC values could be observed when varying the sample by sample amplitude (*α*). Same results were obtained also for the *Fourier*-*based* simulations. This seems to be in contrast with (Røislien et al. 2012), which reports low CMC values obtained from a data-set characterised by small range of motion. Differently from the approach adopted here in which the parameters were varied one at a time, the CMC calculated in (Røislien et al. 2012) accounted for simultaneous variations of offsets, time shift and ROM fluctuations. Looking at the results from the within-subject analysis on the *Fourier*-*based* data, when the same ROM fluctuations were imposed to hip, knee and ankle kinematics, CMC values within the range of ‘excellent similarity’ were obtained for both hip and knee, whereas lower CMCs, which could be classified as ‘very good similarity’, were obtained for the ankle. Thus, CMC is sensitive to the curve shape (Growney et al. 1997; Steinwender et al. 2000), and when different joints are considered, a stratification of CMC values (Garofalo et al. 2009) should be carefully adopted to avoid misinterpretation of the results even if a within-subject comparison is performed. The CMC was also affected by the time shift and offset variations, with some of the latter even causing the coefficients to reach complex values (Table 3), as reported also in (Ferrari, Cutti, Cappello 2010). In that paper complex CMCs were reported even for smaller offsets, most likely due to a simultaneous presence of offset and time shift between the investigated curves, rather than offset only. The data here presented showed low CMC values also when imposing a large time shift between the curves. When dealing with confusing-factors having ranges comparable with the variability of kinematics of healthy subjects, as in *Fourier*-*based* simulation (Table 4), the effect of the time shift on the CMC resulted to be predominant on the effect of the offset. The only exception was found for the CMC-BS of the hip, but it could be ascribed to the highest value of imposed offset (10°) with respect to the other cases. This trend was confirmed by the results obtained from the ‘mixed simulations’ (MS) that produced a decrease in the CMCs. The dependence of CMCs on these confusing-factors highlighted the difficulty of interpreting whether low values of CMC are due to a large offset or a high time shift between the curves. Our findings recommend the CMCs to be interpreted only after having established, at least via a visual inspection of the curves, the presence or absence of large offsets and time shifts.

The LFM yielded three coefficients, which did not vary when changing the ROM of the *sine-curve*. The scaling factor (*a*_1_) reflected the changes in the sample by sample amplitude variations (*α*). This emerged clearly looking at SD − *a*_1_, where null *a*_0_ and *R*^2^ equal to 1 were found. Results in Table 4 (*α* = 5%, WS) showed equal $\overset{¯}{R^{2}}$ and SD − *R*^2^ for different joints, indicating that *R*^2^ is not dependent from the curve shape. Variations of the imposed offset reflected onto the SD − *a*_0_, whereas *a*_1_ and *R*^2^ remained equal to their ideal values. The *a*_0_ represents directly the offset when comparing only two curves (Iosa et al. 2014). However, increasing of the number of curves under investigation led to a mismatch between the obtained ${\overset{¯}{a}}_{0}$ and SD − *a*_0_, and the offset. In fact, the ${\overset{¯}{a}}_{0}$ is always equal to zero even if the offset among curves increased, and the SD − *a*_0_ is only an estimate of the offset variation. The only confusing-factor that invalidated the assumption of a linear relationship between the compared curves was the time shift (*τ*). Indeed, when LFM is adopted in gait studies, the decrease in *R*^2^ should be interpreted as presence of time shift between the curves, and the other coefficients should not be further used. Thus, variations of the scaling factor *a*_1_ cannot be directly interpreted as variations in the ROM fluctuations (*α*). In fact, when *R*^2^ is not equal to 1, the effects of both time shift and ROM fluctuations might be confused. Moreover, SD − *a*_1_ and SD − *a*_0_ obtained for the mixed simulation were equal to those obtained for time shift simulation, despite the range of variations of amplitude variability and offset were the same of those imposed in *α* and *O* simulations. This suggests that the time-shift affects the LFM coefficients more than the other confusing-factors. Hence, *R*^2^ is a measure of the time shift between the compared curves. It can be concluded that LFM separates the effects of the confusing-factors over the three coefficients only when *R*^2^ tends to its ideal value, and the *a*_0_ does not measure the offset but its standard deviation provides only information on the offset variability.

By definition, MAV and RMSD provide an absolute measure of the averaged distances among the curves over the gait cycle. Consistently, their values increased with the increase of all the confusing-factors. From the results in Table 3 (Case 1), these two indices resulted to be strongly related to the range of motion of the curve they were calculated for. However, when the offset was the only imposed variation to the sine-curve (Table 3, case 3), MAV exactly equalled the offset, whereas the range of RMSD values was always equal to the 42% of the offset. When varying only the time shift between curves (*τ*, Table 3, Case 4), MAV and RMSD increased as they were detecting distances due to amplitude variations (*α*) and offsets (*O*). These results allow concluding that MAV and RMSD are representative measures of the averaged distances between the curves only when the time shift can be neglected. In the other cases, indices like SD, Median Absolute Deviation (MAD) and Maximum Difference (MD) (Benedetti et al. 2013), calculated on joint kinematics at specific instants of the gait cycle should be preferred.

## Conclusions

This study illustrated how to apply and interpret the investigated repeatability and reproducibility indices. In particular, the ROM of the curves was proved to not influence the CMC or the LFM coefficients; conversely, the CMC resulted sensitive to the curve shape, leading to possible misinterpretations of the results when comparing data from different joints. Moreover, values of the CMC became meaningless when large offset and time shift occur, as it reaches complex values. Therefore, given a set of data, the LFM should be used to assess its repeatability and reproducibility. In fact, SD − *a*_1_, SD − *a*_0_ and *R*^2^ provide information on amplitude variability, offset and time shift, respectively, and a value of *R*^2^ approaching to 1 leads to the conclusion that time shift might be neglected. Alternatively, MAV and RMSD might also be used as measurements of the data dispersion, but keeping in mind that they would not be able to univocally discriminate among the different confusing-factors. When time shift occurs, an assessment of data repeatability and reproducibility evaluated on summary metrics (e.g. kinematics calculated at initial contact, or toe-off) is likely to be preferred to the here investigated indices.

## Supplementary Material

TBBE_1426496_Supplementary_materials.docx

## Supplementary data

This supplementary data for this article can be accessed here https://doi.org/10.1080/23335432.2018.1426496
