# Supplementary material for: How to choose and interpret similarity indices to quantify the variability in gait joint kinematics
Source: Int Biomech. 2018 Feb 1;5(1):1–8. doi: 10.1080/23335432.2018.1426496 (PMC7857465; doi:10.1080/23335432.2018.1426496)
Supplement: TBBE_1426496_Supplementary_materials.docx [file TBBE_A_1426496_SM7925.docx]

**How to choose and interpret similarity indices to quantify the variability in gait joint kinematics – Supplementary materials**

**Appendix A: Examples of the generated sine-curve data**

Groups of five curves were generated from the following mathematical model, where$j$refers to the stride, and $t$ to the time point:

| **Range of Motion (*ROM*)** | **ROM variability (*α*)** |
| --- | --- |
| 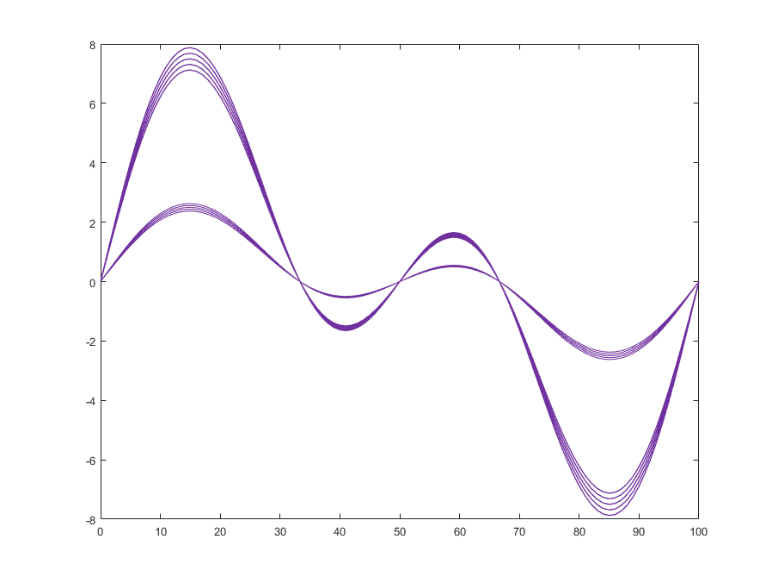 | 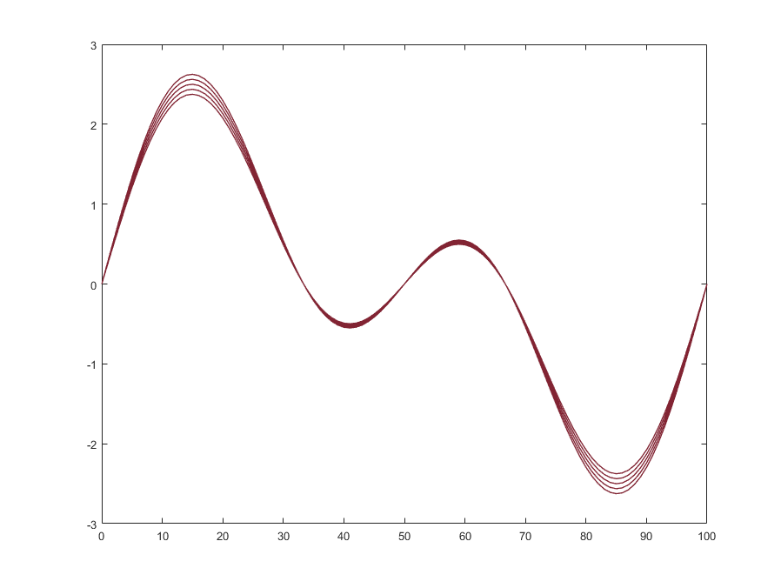 |
| **Offset (*O*)** | **Time shift (τ)** |
| 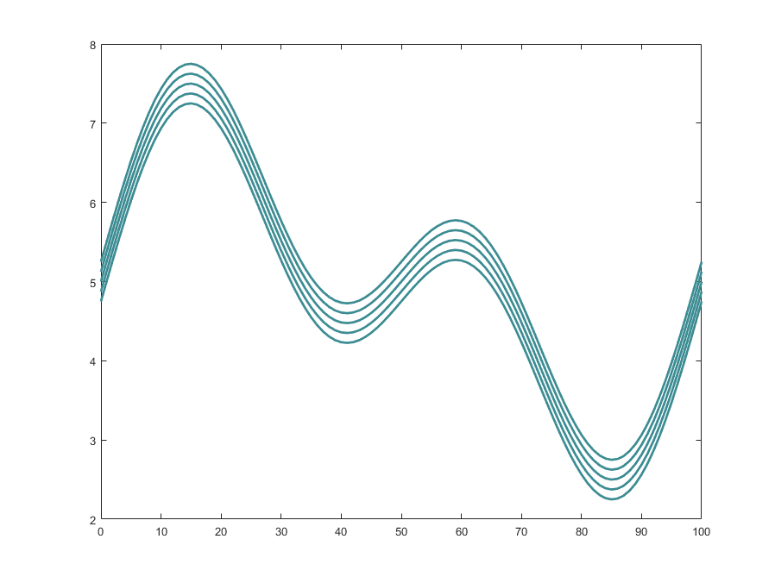 | 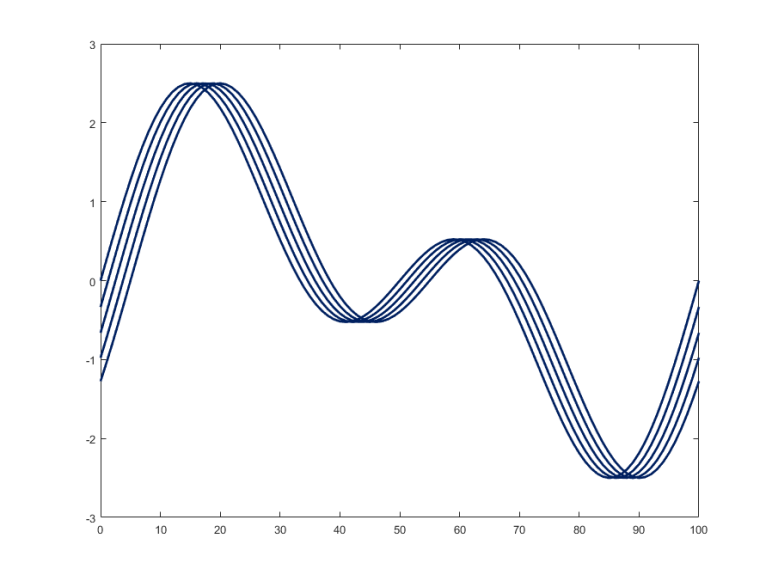 |

**Appendix B: Procedure to simulate the Fourier-based data**

Starting from the averaged sagittal hip, knee and ankle kinematics obtained from the experimental data, a Fourier decomposition was performed to obtain the coefficients $A_{0}$, $A_{k}$ and $B_{k}$ according to the following formula:

To simulate each confusing-factor on the reconstructed curve, the Fourier’s coefficient ($A_{0}$, $A_{k}$ and $B_{k}$) were modified as follows:

| **ROM variability (*α*)** | |
| --- | --- |
| - A random number (*x*) was generated within the imposed range of variation of α *(%_ROM_)* (see Table 2 in the manuscript); - To obtain α, *x* was multiplied by the range of motion of the considered curve and divided by 100; - The new Fourier’s coefficients were: $A_{0}^{*}=A_{0}$ $A_{k}^{*}=\left(1+\alpha\right)\cdot A_{k}$ $B_{k}^{*}=\left(1+\alpha\right)\cdot B_{k}$ - The curve$y^{*}(t)$ was then reconstructed. | 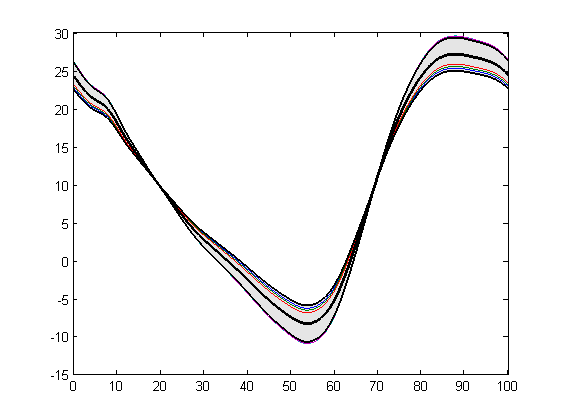 |
| **Offset (*O*)** | |
| - A number (Off) was fixed as the double of the chosen percentage of offset O *(%_ROM_)* (see Table 2 in the manuscript); - A random number (*O*) was generated within the range $\left[-\frac{\text{Off}}{2};\frac{\text{Off}}{2}\right]$; - The new Fourier’s coefficients were: $A_{0}^{*}=A_{0}+O$ $A_{k}^{*}=A_{k}$ $B_{k}^{*}=B_{k}$ - The curve$y^{*}(t)$ was then reconstructed. | 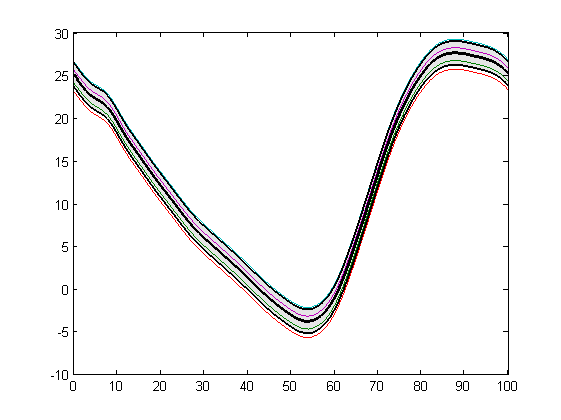 |
| **Time shift (τ)** | |
| - A random integer number (τ) was generated according to the chosen value of percentage of the gait cycle τ *(%_GaitCycle_)* (see Table 2 in the manuscript); - The Fourier’s coefficients were left unvaried; - The τ was then imposed to shift the curves over the gait cycle. - The curve$y^{*}(t)$ was then reconstructed. | 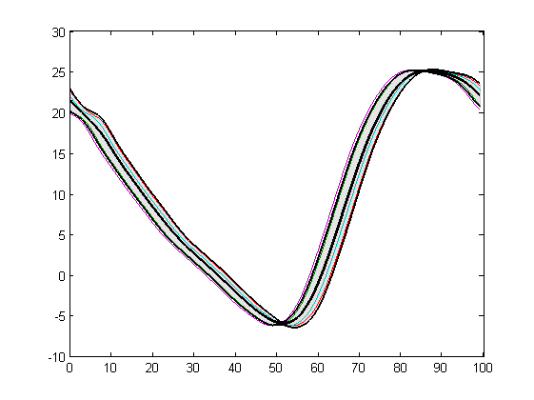 |
| **Mixed (MS)** | |
| - A random number (*x*) was generated within the imposed range of variation of α *(%_ROM_)* (see Table 2 in the manuscript); - To obtain α, *x* was multiplied by the range of motion of the considered curve and divided by 100; - A number (Off) was fixed as the double of the chosen percentage of offset O *(%_ROM_)* (see Table 2 in the manuscript); - A random number (*O*) was generated within the range $\left[-\frac{\text{Off}}{2};\frac{\text{Off}}{2}\right]$; - A random integer number (τ) was generated according to the chosen value of percentage of the gait cycle τ *(%_GaitCycle_)* (see Table 2 in the manuscript); - The new Fourier’s coefficients were: $A_{0}^{*}=A_{0}+O$ $A_{k}^{*}=\left(1+\alpha\right)\cdot A_{k}$ $B_{k}^{*}=\left(1+\alpha\right)\cdot B_{k}$ - The τ was then imposed to shift the curves over the gait cycle; - The curve$y^{*}(t)$ was then reconstructed. | 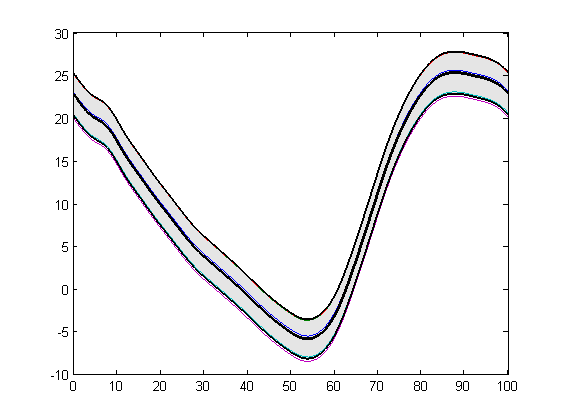 |

Five curves were generated for each case of confusing‑factor, simulating five strides of a subject. Then, a Monte Carlo procedure was performed to run 1000 simulations for each group of five curves. Eventually, the repeatability and reproducibility indices were calculated on each set of curves. The curve decomposition and reconstruction was performed using the Matlab functions *Fseries* and *Fseriesval* available at [www.mathworks.com](http://www.mathworks.com) (The MathWorks, Inc. – Natick, MA, USA).
